# Palliative care awareness amongst religious leaders and seminarians: a Nigerian study

**DOI:** 10.11604/pamj.2017.28.259.14010

**Published:** 2017-11-23

**Authors:** Adewale Ismahil Badru, Kehinde Kazeem Kanmodi

**Affiliations:** 1 Emergency Department, University College Hospital, Ibadan, Nigeria; 2 Cephas Health Research Initiative Inc, Ibadan, Nigeria; 3 Department of Dental and Maxillofacial Surgery, Usmanu Danfodiyo University Teaching Hospital, Sokoto, Nigeria; 4 Community Health Officers Training Programme, Usmanu Danfodiyo University Teaching Hospital, Sokoto, Nigeria

**Keywords:** Nigeria, palliative care, awareness, religious leaders, seminarians

## Abstract

**Introduction:**

There exists scanty literature on the awareness of Nigerians towards palliative care. This study was conducted to determine the level of awareness of religious leaders and seminarians in Ibadan, Nigeria, on palliative care.

**Methods:**

Data obtained from a cross-section of 302 religious leaders and seminarians in the metropolitan city of Ibadan, Nigeria, were used in this research. Study tool was a self-administered questionnaire which obtained information from the participants about their bio-data and knowledge of palliative care. Data analysis was done using the SPSS version 16 Software.

**Results:**

The mean age of the respondents was 28.99 years, with 55.6% of them being within the age range of 21 to 30 years. The majority (94%) were males, 68.5% were single and 79.8% were seminarians. Only 31.8% have heard of palliative care before, 12.6% knew a health facility where palliative care is rendered in Nigeria, while 21.9% knew that chaplains are members of the palliative care team.

**Conclusion:**

The level of awareness of palliative care among religious leaders and seminarians in Ibadan, Nigeria, is low. There exists the need to educate Nigerian religious leaders and seminarians on palliative care.

## Introduction

Palliative care, according to the World Health Organization (WHO), can be defined as an approach that improves the quality of life of patients and their families facing the problems associated with life-threatening illness, through the prevention and relief of suffering by means of early identification and impeccable assessment and treatment of pain and other problems, physical, psychological and spiritual [1]. Palliative care is a multidisciplinary patient-centred care and it involves the contributions from a team of professionals comprising of chaplains, nurses, oncologists, social workers, anaesthetists, radiologists and other health care officers [2]. This team provide social, medical, spiritual and emotional care to these patients, in order to ensure that they enjoy a good quality of life [1, 2]. Historically, modern palliative care services came into existence about five decades ago, as a result of the rising prevalence of the burden of life-threatening chronic diseases (e.g. cancer, hypertension and diabetes mellitus) coupled with the increasing health needs of the population living with such diseases [3, 4]. The first modern palliative hospice in the world, St. Christopher's Hospice, was founded in 1967 in the United Kingdom by Cicely Saunders [3]. In 2007, the first Nigerian public palliative hospice was officially opened on 19^th^ July, 2007, at the University College Hospital (UCH), Ibadan, Nigeria [5]. Thereafter, another five public palliative hospices were established in other geographic locations within the country. Quite a number of studies had reported awareness rates about palliative care among the Western populations [6-9]. For instance, at least 4 in 10 adults were found to be aware of “palliative care” in different surveys conducted in Australia, Canada, Ireland and Scotland [6-9]. However, only scanty studies are available on the public awareness rates about palliative care in Nigerian populations and most of these studies reported awareness rates among health workers [10-13]. This study aimed to determine the level of palliative care awareness amongst religious leaders and seminarians in the metropolitan city of Ibadan, Nigeria. The religious leaders were considered to play important roles in palliative care. These roles include visiting palliative patients at hospices and rendering spiritual and emotional care to the palliative patients and their families [2, 14]. The seminarians are the future religious leaders; they are individuals undergoing religious training in preparation for the leadership roles they will occupy in future. This kind of study conducted among these population groups is of high significance as it provided information on their knowledge of palliative care.

## Methods

This was a cross-sectional study conducted among religious leaders and seminarians in the ancient city of Ibadan, Oyo State, Nigeria. This city is the capital of Oyo State and she is also the largest city in West Africa [15]. Ibadan is a home to many of the major Nigerian religious institutions [15]. This city is also the home to the first public palliative hospice in Nigeria [5]. This study was conducted under compliance with the ethical guidelines of the Helsinki Declaration. Permission to carry out this study was officially obtained from the Ministry of Education, Ibadan, Nigeria (Ref: INS.2959/80). Permission to collect data was also obtained from the heads of the participating institutions. All participants were informed about the aims and objectives of the study. They were also informed that their participation is voluntary and confidential. Only those that gave verbal informed consent were recruited for the study. None of the study participants was asked to provide any information about their personal identity; hence their participation was strictly confidential. The study tool was an anonymous well-structured questionnaire. The questionnaire had two sections: section A; and section B. Section A obtained information from the participants about their demographic data such as: age, gender, tribe, religion, level of education and the religious position occupied (religious leader/seminarian). Section B obtained information about the participants: awareness of palliative care, using a “yes/no/I am not sure” options as a response to the question “Have you heard of palliative care before?“; knowledge of the meaning of “palliative care” using a five-option response to the question “What is the meaning of palliative care?” (Only one of the provided options gave the lay man description of “ palliative care” as “ the management of pain in dying patients and patients with chronic diseases (e.g. cancer) [6], while the other four options gave wrong descriptions of “ palliative care”); and knowledge of the members of the palliative care team.

Sample size was determined using the Leslie formula [16]:

“N = Z^2^ P(1 – P)/T^2^”

Where N is the sample size, Z is the level of significance that corresponds to the 95% confidence level (that is, Z = 1.96), P is the prevalence taken as 76.3% [7] and T is the tolerance error (T = 0.05). The calculated sample size was 278. Using cluster sampling technique, a total of ten religious institutions in Ibadan, Nigeria, were chosen for this study. The participating institutions were: two community central mosques, two seminaries; two university departments of Religious Studies; the headquarters of one of the leading national religious organization in the country; and three big local churches. The study participants were selected based on two criteria: (1) being a: religious leader; or a student of Religious Studies at the institutions or at the seminaries selected for the study; (2) willingness to participate in the study. Those that were studying in the religious institutions of learning, and with the aim of becoming a religious leader in future, were grouped as seminarians. Those that were already occupying leadership roles in religious institutions (i.e. mosques, churches and missionary organizations) were grouped as religious leaders. Data collection was carried out from October to December, 2014. The participants were approached at their institutions. The aims and objectives of the study were clearly explained to them. They were also informed that their participation is voluntary and confidential. Verbal informed consent was obtained from each participant that volunteered to participate in the study. A total of 350 participants were given questionnaires to fill, of which only 311 returned their questionnaires filled. During the data cleaning process, 9 out of these 311 questionnaires were discarded due to incomplete data; hence data of 302 respondents was finally used for this study. Data analysis was done using the SPSS version 16 software. Frequency distributions of all variables were determined and illustrated using tables.

## Results

The mean age of the 302 respondents was 28.99 years, with 55.6% of them being within the age range of 21 and 30 years. The majority (94.0%) were males, 68.5% were single, 49.7% were from the Yoruba tribe, 47.4% were Catholic and 77.5% had their education to the bachelor degree level (Table 1). Sixty one (20.2%) respondents were religious leaders, while the remaining respondents (79.8%) were seminarians. Just 96 (31.8%) respondents categorically reported to had ever heard of palliative care (Table 2), out of which only 75% (72/96) of them knew its actual meaning (Table 3). Not all of these aforementioned 96 respondents had a full knowledge of the members of the palliative care team; the top three professional members known by them were nurses (95.8%), physicians (94.6%), and psychologists (94.6%) (Table 4). Furthermore, only 35.4% of them (34/96) knew of a health facility in Nigeria where palliative care is rendered (Table 5).

**Table 1:** Socio-demographic features of respondents

Characteristics (n=302)	N	%
**Gender**		
Male	284	94.0
Female	17	5.6
Not specified	1	0.3
Mean age^+^	28.99	
**Marital status**		
Single	207	68.5
Married	92	30.5
Not specified	3	1.0
**Religion**		
Islamic	23	7.6
Catholic	143	47.4
Protestant	60	19.9
Pentecostal	75	24.9
Tribe		
Yoruba	150	49.7
Igbo	48	15.9
Hausa	5	1.7
Others	99	32.8
**Level of education**		
Secondary	10	3.3
OND	10	3.3
HND	7	2.3
NCE	1	0.3
Bachelor	234	77.5
PGD	1	0.3
Master	30	9.9
Doctorate	10	3.3

n=Total number of respondents; N=Number of respondents in each socio-demographic category; OND=Ordinary National Diploma; HND=Higher National Diploma; NCE=Nigerian Certificate in Education; PGD=Postgraduate Diploma. ‪Age in years

**Table 2:** Awareness of respondents on palliative care

Response	Have you heard of palliative care before? (n=302)	
	N	%
Yes	96	31.8
No	166	55.0
I am not sure	34	11.3
No response	6	2.0

N = Total number of respondents; N = number of respondents in each category of responses

**Table 3:** Knowledge of the meaning of palliative care among the 96 respondents that claimed awareness of palliative care

Responses (n=96)	N	%
Family planning through the use of contraceptives, vasectomy	4	4.2
Welfare of brethren in the same religion	11	11.5
Maintenance and repair of hospital facilities	1	1.1
Management of pain in dying patients, and patients with chronic diseases	72	75.0
Provision of security for religious leaders during religious crisis	2	2.1
Prevention and control of haemorrhagic fevers like Ebola, Lassa, Dengue fever	5	5.2

n=total number of respondents that claimed to know the meaning of palliative care; N=Total number of respondents in each category

**Table 4:** Knowledge of the members of the palliative care team by the 96 respondents that reported to have heard of palliative care

Members of the palliative care team (n=96)	Yes	%
Chaplains	66	68.8
Anaesthesiologists	67	69.8
Oncologists	55	57.3
Nurses	92	95.8
Physiotherapists	84	87.5
Psychologists	87	94.6
Physicians	87	94.6
Social workers	61	63.5

n=Total number of respondents that have ever heard of “palliative care”

**Table 5:** Knowledge of a Nigerian palliative care centre by the 96 respondents that reported to have heard of palliative care

Response (n=96)	Do you know any location where palliative care is rendered in Nigeria?	
	N	%
Yes	34	35.4
No	58	60.4
No response	4	4.2
Total	96	100.0

N = Total number of respondents that have ever heard of “palliative care”; N = Total number of respondents in each category

## Discussion

The religious leaders are known to be key spiritual leaders in the Nigerian society. They are primarily shouldered with the duty of providing spiritual care, irrespective of other administrative roles they also perform. One of the peculiar duties of a religious leader is to pray for individuals with diseased conditions. As a matter of fact, religious leaders, particularly the chaplains, are eligible members of a standard palliative care team, and their role is to provide spiritual and emotional care to palliative patients [2]. This study provided information on the awareness rate of religious leaders and seminarians in Ibadan, Nigeria, on palliative care. The rationale for including the seminarians in this survey was because they are the future religious leaders and they are also actively engaged in religious activities in the Nigerian society, even when they are still undergoing training in the seminary. To the best of the authors' knowledge, this is the first research paper on awareness of palliative care among this population group from the Nigerian setting. In this study, we observed that many of our respondents were unaware of palliative care. Many of them also lacked knowledge of the presence of a palliative hospice in Nigeria. This awareness rate (31.8%) among our respondents is relatively very low when compared to that reported among public populations in the Western countries [7]. For example, palliative care awareness rate of 76.3%, 75% and 49% were recorded among Irish, Canadian, and Scottish populations respectively [8-10]. From the authors' point of view, the low awareness rate recorded among the respondents in this study might be due to these factors: palliative care is a relatively new area in the Nigerian health care system [5]; only little efforts had been made on public awareness about palliative care in the Nigerian society [13]. This study has its limitations. Only the religious leaders that were literates were surveyed in this study; hence data from those that were not able to read and write in English were not captured in this study. This study also did not capture religious leaders and seminarians in other Nigerian cities; hence the need to conduct a bigger study in future.

## Conclusion

This study reveals a very low awareness rate of palliative care among religious leaders and seminarians in Ibadan, Nigeria. The authors would like to recommend the use of educational programmes to enlighten this population group, and even the public-at-large, on palliative care.

### What is known about this topic

- Palliative care is an emerging field in the Nigerian health sector;
- Awareness on palliative care had been studied among some Nigerian health professionals;
- Awareness on palliative care among the general Nigerian populations is understudied.

### What this study adds

- Awareness rate of palliative care amongst religious leaders and seminarians in Ibadan, Nigeria, was generally low;
- This study recommends public education on palliative care among the lay Nigerian populations.

## Competing interests

The authors declare no competing interests.
